# Development of a Surface Plasmon Resonance Biosensor for Real-Time Detection of Osteogenic Differentiation in Live Mesenchymal Stem Cells

**DOI:** 10.1371/journal.pone.0022382

**Published:** 2011-07-27

**Authors:** Yi-Chun Kuo, Jennifer H. Ho, Ta-Jen Yen, How-Foo Chen, Oscar Kuang-Sheng Lee

**Affiliations:** 1 Institute of Clinical Medicine, National Yang-Ming University, Taipei, Taiwan; 2 Stem Cell Research Center, National Yang-Ming University, Taipei, Taiwan; 3 Graduate Institute of Clinical Medicine, Taipei Medical University, Taipei, Taiwan; 4 Center for Stem Cell Research, Taipei Medical University-Wan Fang Medical Center, Taipei, Taiwan; 5 Department of Ophthalmology, Taipei Medical University-Wan Fang Medical Center, Taipei, Taiwan; 6 Department of Material Science and Engineering, National Tsing Hua University, Hsinchu, Taiwan; 7 Institute of Biophotonics, National Yang-Ming University, Taipei, Taiwan; 8 Department of Orthopaedics and Traumatology, Taipei Veterans General Hospital, Taipei, Taiwan; University of South Florida, United States of America

## Abstract

Surface plasmon resonance (SPR) biosensors have been recognized as a useful tool and widely used for real-time dynamic analysis of molecular binding affinity because of its high sensitivity to the change of the refractive index of tested objects. The conventional methods in molecular biology to evaluate cell differentiation require cell lysis or fixation, which make investigation in live cells difficult. In addition, a certain amount of cells are needed in order to obtain adequate protein or messenger ribonucleic acid for various assays. To overcome this limitation, we developed a unique SPR-based biosensing apparatus for real-time detection of cell differentiation in live cells according to the differences of optical properties of the cell surface caused by specific antigen-antibody binding. In this study, we reported the application of this SPR-based system to evaluate the osteogenic differentiation of mesenchymal stem cells (MSCs). OB-cadherin expression, which is up-regulated during osteogenic differentiation, was targeted under our SPR system by conjugating antibodies against OB-cadherin on the surface of the object. A linear relationship between the duration of osteogenic induction and the difference in refractive angle shift with very high correlation coefficient was observed. To sum up, the SPR system and the protocol reported in this study can rapidly and accurately define osteogenic maturation of MSCs in a live cell and label-free manner with no need of cell breakage. This SPR biosensor will facilitate future advances in a vast array of fields in biomedical research and medical diagnosis.

## Introduction

Dramatic progress in the biological understanding and the potential clinical use of mesenchymal stem cells (MSCs) has been made in recent years. MSCs have been initially identified in bone marrow stroma as non-hematopoietic stem cells which are capable of differentiation into tissues of mesodermal origin, such as osteoblasts, adipocytes, chondrocytes, tenocytes, and hepatocytes [1], [2], [3], [4], [5], [6]. Due to their multi-lineage differentiation potentials, many pre-clinical studies with tissue engineering approaches are currently under investigation [7], [8], [9]. Previously, we have established a platform to isolate and to expand single cell-derived, clonally expanded MSCs from human bone marrow and umbilical cord blood through negative immune-selection and limiting dilution [6], [10]. These single cell-derived hMSCs are highly homogenous in morphology; and possess a high capacity of *in vitro* expansion and multi-lineage differentiation.

Osteoblasts, which are progenies of MSCs, are bone-forming cells and play an important role in the homeostasis of the skeletal system [11], [12], [13]. Current strategies for the differentiation of stem cells commonly include induction with mechanical or chemical stimuli. To evaluate the maturation of osteogenic differentiation of hMSCs during these processes, histochemical and molecular biological methods such as alkaline phosphatase (ALK-p) staining, von Kossa staining, Western blot, and reverse transcription polymerase chain reaction (RT-PCR), are commonly used [14], [15], [16]. However, all these traditional methods are time-consuming with tedious process and can only provide semi-quantitative or non-quantitative data except for the real-time RT-PCR. Moreover, the conventional methods to detect the extent of osteogenic differentiation require cell lyses or fixation, which causes cell death and makes continuous analysis on the same cell impossible.

Surface plasmon resonance (SPR) biosensors dedicated to biomolecular dynamics and recently to cell analysis have generated tremendous interest in developing new tools for both diagnostic and research purposes. This technique is a surface-sensitive method of increasing interest for bio-analysis as it allows label-free and real-time analysis of biomolecule interactions on functionalized surfaces [17], [18], [19], [20], [21], [22]. The primary goals for the development of this technique is to establish a method with rapid live cell analysis, high throughput, and small sample volumes [23]. For this purpose, selection of a proper surface marker for osteogenesis is imperative. The cell transmembrane protein, OB-cadherin, firstly cloned in 1994 [24], [25], is known to selectively express in osteoblastic cell lines, precursor cell lines of osteoblast, and primary osteoblastic cells [26]. The purpose of this study is to investigate whether the SPR technique can be used as a live cell sensor to accurately define the different stages of osteogenic maturation in live cells by detecting the expression of OB-cadherin on cell surfaces.

## Methods

### 2.1 Culture maintenance and expansion

For studies involving human tissues we obtained Institutional Review Board approval of Taipei Veterans General Hospital on March 24th, 2010 and written patient informed consent. Bone marrow was collected from healthy young donors during fracture surgery after Institutional Review Board approval and informed consent. Mononuclear cells from the bone marrow were isolated and MSCs were purified with negative immuno-selection and limiting dilution as previously described [10]. SaOS2 [27] is an OB-cadherin expressing cell line and is used as a positive control. Hep3B is a human hepatoma cell line [28] and served as an OB-cadherin non-expression control. Expansion medium for MSCs consists of a commercially available medium (MesenPro, Gibco, Grand Island, NY) supplemented with 100 U penicillin, 1000 U streptomycin, and 2 mM L-glutamine (Gibco). Expansion medium for SaOS2 and Hep3B consists of Iscove's modified Dulbecco medium (IMDM; Gibco, Grand Island, NY) and 10% fetal bovine serum (FBS; Hyclone, Logan, UT) supplemented with 100 U penicillin, 1000 U streptomycin, and 2 mM L-glutamine (Gibco).

### 2.2 Osteogenic differentiation

To induce osteogenic differentiation, MSCs were treated with osteogenic medium for 15 days with medium changes every 3 to 4 days. Osteogenesis was analyzed every 3 days. Osteogenic medium consists of IMDM supplemented with 0.1 µM dexamethasone (Sigma-Aldrich, St Louis, MO), 10 mM β-glycerol phosphate (Sigma-Aldrich), and 0.2 mM ascorbic acid (AsA; Sigma-Aldrich).

### 2.3 Cytochemical staining of osteogenic differentiation

To analyze osteogenesis, cells were rinsed twice with PBS, fixed with 3.7% formaldehyde for 20 minutes, and washed with distilled water. Alkaline phosphatase (Alk-P) histochemical stain was performed using the nitroblue/BCIP solution (Roche Molecular Biochemicals, location?) based on the manufacturer's instructions. Mineralization matrix was analyzed with von Kossa staining using 1% silver nitrate (Sigma-Aldrich) under UV light for 45 minutes, followed by 3% sodium thiosulfate (Sigma-Aldrich) for 5 minutes, and then counterstained with van Gieson (Sigma-Aldrich) for 5 minutes.

### 2.4 Antibody coating of Au chip

Au chip was immersed in 1 mM 11-MUA in 75% alcohol for 12 hr, followed by immersion in 2 mM∶5 mM EDC/NHS for one hour. The chip was then coated with 0.005 ug/ml protein G in PBS for one hour, and bound with 0.05 ug/ml mouse IgG anti-human OB cadherin (Abnova) or mouse IgG (sigma) in PBS for one hour, and finally blocked in 1 mM BSA.

### 2.5 Surface plasmon resonance system

**Figure 1:**
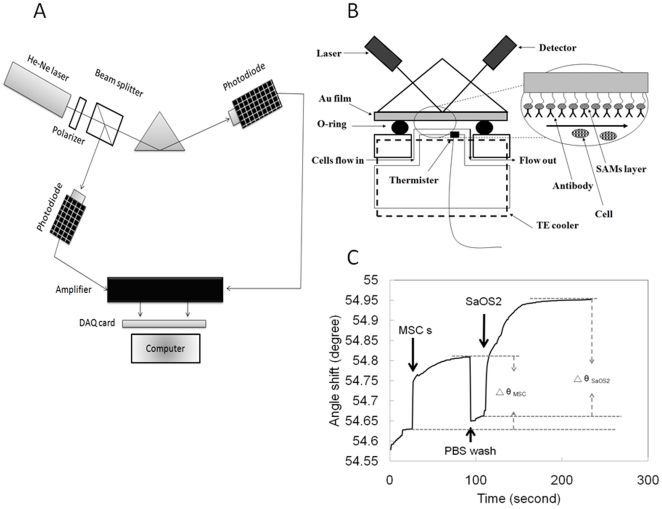
SPR setups and experimental procedures. (A) Schematic illustration of the SPR device: The He-Ne laser penetrated through a polarizer and a beam splitter, which split the beam 50/50. One was detected by a photodiode and the other one was coupled by a prism to generate Surface Plasmon Wave on the Au chip in which the angle shift was detected by a photodiode. (B) The OB-cadherin expressing cells flowing into the chamber were captured by the Au chip pre-coated with OB-cadherin antibodies, which changed the angle of the reflected laser beam. (C) A typical graphical data from SPR measurements.

The experimental setups for the evaluation of osteogenic differentiation of MSCs are schematically illustrated in Fig. 1. The setup combines the platforms of the biosensor for the excitation of surface plasmon polaritons (Fig. 1A) and a fluidic cell chamber (Fig. 1B). As illustrated in Fig. 1A, a Helium–Neon laser of wavelength 632.8 nm was used as the light source to excite surface plasmon polaritons. The extinction ratio of beam polarization was enhanced to 1000∶1 through the aid of a polarizer in front of the laser. This polarizer was oriented to let only the p-wave pass. A beam splitter (BS) was used to separate the laser beam into two beams. One p-wave was reflected to a detector for normalization of the laser power instability and the other was transmitted to excite surface plasmon polaritons. Since the dispersion characteristic of surface plasmon polaritons forbids a p-wave in free space couple to a surface plasmon wave due to the smaller spatial phase of the wave in free space. A prism made of SF11 adds an extra spatial phase on the laser beam through its high refractive index, n = 1.78, to match the dispersion relation of the surface plasmon polaritons. For a practical reason, an Au thin layer with thickness 47.5 nm was coated on a glass slide made of SF11 as the platform of the surface plasmon polaritons. Matching oil was applied between the prism and the glass slide to avoid multi-reflection occurring between them. The energy coupling between the laser beam in a free space and the surface plasmon polaritons bound on the Au-dielectric interface was measured through the reflection of the laser beam from the Au chip. This reflection served as the indicator of the coupling or excitation efficiency. This excitation efficiency was optimized when the incident angle of the laser beam is properly adjusted. This optimized incident angle, known as the resonance angle, is determined by the refractive index of any material contacting the Au chip and the wavelength of the excitation light beam as well. In this system, the incident angle of the laser beam was controlled by a motorized rotation stage through a controller, and a silicon photodetector was controlled accordingly by another motorized rotation stage to measure the power of the reflected laser beam. These two rotation stages (Sigma Koki, SGSP-120YAW) controlled by the two-axis controller (Sigma Koki, SHOT-202) have the angular resolution of 2.5×10^−3^ degrees. The intensity of the reflected beam as a function of the incident angle was recorded by a computer via 16-bit A/D converter (Adventech PCI-1716). A cell chamber equipped with a fluidic channel was constructed on the SPR platform to provide the living cells for testing and to flow deionized (DI) water for stabilizing and for washing unbound cells. The temperature of the cell chamber was stabilized at 37°C by a TE cooler placed on the top of the chamber. The temperature fluctuation is less than 0.1°C through a temperature controller.

### 2.6 Experimental procedures

As depicted in Fig. 1B and Fig. 1C, undifferentiated MSCs, MSCs under osteogenic induction for different days, Hep3B and SaOS2 cells were individually mixed with PBS and injected into cell chambers with Au chips and subject to three different surface treatments: one without antibody coating, one with mouse IgG antibody coating (non-specific binding control), and one with pre-coated with OB-cadherin antibody. The flow rate and cell number was controlled at 1000 cells/min. The angle shifts of captured cells on Au chips subject to the three different surface treatments were measured and recorded by the SPR system operating in the angular interrogation mode as previously described. After the angle increased to the level of saturation, flushing with PBS was performed to wash out the cells until the angle shift returned to the initial level. The subsequent population of cells was then injected on the Au chip for further measurements.

### 2.7 Quantitative real-time PCR analysis

Total RNA was isolated from 3×10^5^ cells using Trizol (Invitrogen) or a commercially available kit (RNA easy minikit, Qiagen, Courtaboeuf, France). We reverse transcribed the messenger RNA to complementary DNA using reagents (Genemark Technology, Taiwan) according to the manufacturer's instructions. Quantitative real-time PCR analysis was performed and cDNA was amplified at 95°C for 60 seconds, 56°C for 45 seconds, and 72°C for 60 seconds for 40 cycles, after initial denaturation at 95°C for 5 minutes using an ABI Step One Plus Real Time PCR System. The specific primer sequences are 5′-AAACAGCCTGGCTCAACATC-3′ (forward) and 5′-CTTCCTGATGCCGATTGTG-3′ (reverse) for OB-cadherin; 5′-AGCCACATCGCTCAGACAC-3′ (forward) and 5′-GCCCAATACGACCAAATCC-3′ (reverse) for GAPDH.

### 2. 8 Western blot analysis

10^6^ cells were collected and resuspended in 200 µl Extraction Buffer I and 1 µl protease inhibitors cocktail (Subcellular proteome extraction kit, Merck). Cell lysate was incubated on ice for 10 min. with gentle agitation and then centrifuged at 1000 g at 4°C for 10 min. The supernatant was collected, and remixed in 200 µl Extraction Buffer II and 1 µl protease inhibitors cocktail (Subcellular proteome extraction kit, Merck KGaA). Cell lysate was incubated on ice for 30 min. with gentle agitation and then centrifuged at 6000 g at 4°C for 10 min. The supernatant was collected and protein concentration was measured by Bradford assay (Bio-Rad, Hercules, CA). 10 µg/well of protein was separated on 10% sodium dodecyl sulfate/polyacrylamide gel electrophoresis and blotted onto a polyvinylidene difluoride membrane (GE Healthcare Bio-Sciences, Piscataway, NJ). Nonspecific bindings were blocked by 5% skim milk in TBST buffer (50 mmol/L Tris-HCl, 150 mmol/L NaCl, 0.1% Tween 20, pH 7.4). The membrane was sequentially hybridized with the primary anti-OB cadherin antibody (1∶2000, Abcam) and with horseradish peroxidase–conjugated secondary antibody (1∶6000, GE Healthcare Bio-Sciences) at room temperature for 1 hour. Washes were performed between incubations. Protein intensity was resolved with enhanced chemiluminescence reagent (PerkinElmer, Wellesley, MA).

## Results

**Figure 2:**
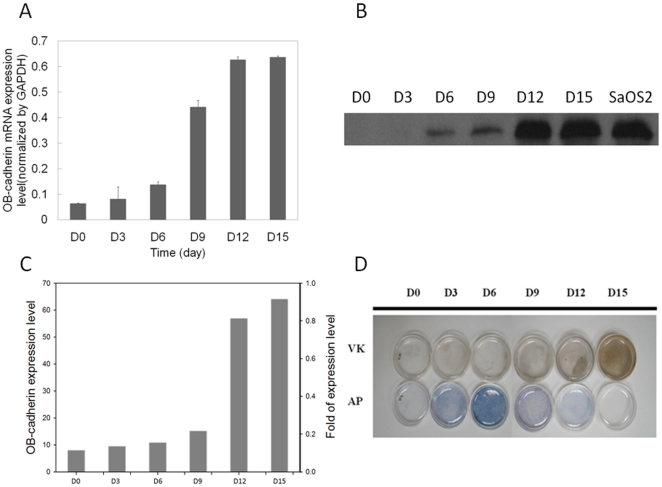
Molecular biology detection of MSC-osteoblast differentiation. (A) Real-time PCR of OB-cadherin expression. (B) Western blot of OB-cadherin expression during osteogenic induction of MSCs. SaOS2 served as positive control. (C) The OB-cadherin expression level intensity analyzed by semi-quantitative method and was normalized to the intensity of SaOS2. (D) Alkaline phosphatase (AP) staining and von kossa (VK) staining.

**Figure 3:**
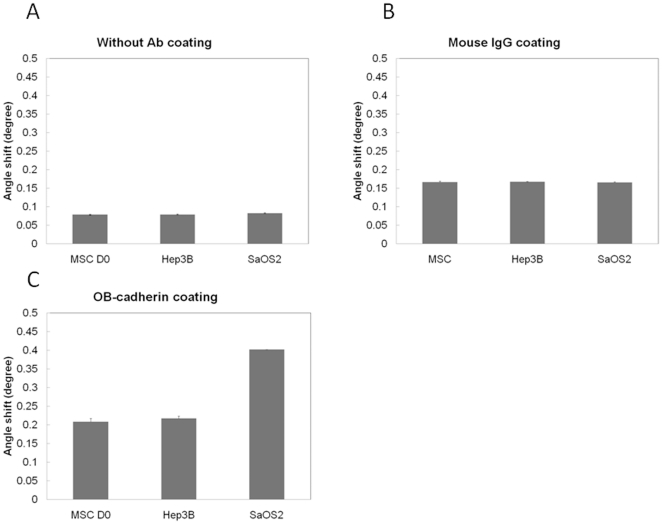
Resonance characteristics of antibody–antigen reaction on Au coated chips. (A) Test control of antibody non-coating system and injected with SaOS2, Hep3B and MSCs onto Au chip. (B) Test control of antibody coating system by injecting SaOS2, Hep3B and MSCs onto mouse IgG pre-coated Au chip. (C) The verification and stability test of OB-cadherin antibody-antigen binding by injecting SaOS2, Hep3B and MSCs.

The mRNA and protein expression level of OB-cadherin increased following the maturation of MSCs during osteoblast differentiation (Fig. 2A and Fig. 2B). The OB-cadherin protein expression level was measured by a semi-quantitative assay in which the relative amount of immunoreactive bands on Western blot film was quantified as arbitrary units using a computerized software program (LabWorks 4.6; UVP Inc., Cambridge, UK) and was normalized to the intensity of SaOS2 (Fig. 2C). The result demonstrated an increase in OB-cadherin expression level as the induction period increased. In addition, successful induction of osteogenic differentiation was confirmed by alkaline phosphatase (AP) and von Kossa staining (Fig. 2D). Since the expression of the membrane molecule OB-cadherin is up-regulated during maturation of osteoblasts, we therefore used it as a marker to analyze the extent of differentiation by quantifying it under SPR system. In order to eliminate non-specific binding of cells to Au chip and false positives, SaOS2 and Hep3B cell lines were used as positive and negative controls for the expression of Ob-cadherin respectively. Furthermore, the Au chip without antibody pre-coating or with mouse IgG antibody pre-coating were used as control groups to eliminate non-specific binding of cells onto Au chip and IgG antibodies. DI water was first injected into the cell chamber for stabilization, followed by PBS as a baseline control. After the injection of PBS, cells were injected for measurements. The true angle shift of each cell line was evaluated by subtracting the resonance angle in the corresponding test baseline from the SPR resonance angle of bound and unbound cells. For the non-coating control groups, SaOS2, Hep3B, and MSCs were injected into the cell chamber without antibody pre-coated on the Au surface. As expected, all the tested cell lines showed no significant difference on bare Au chips because there is no antibody layer to perform specific binding (Fig. 3A). For the antibody coating control groups, the anti-OB cadherin and mouse IgG antibodies were first immobilized on the sensor chips and bathed in a concentration of 10^3^ cells/ml PBS by the self-assembly monolayer (SAM) coating method. The amount of resonance angle shift after injecting the SaOS2, Hep3B, MSCs to the cell chamber with mouse IgG also showed no significant difference because there is no specific cell-antibody binding (Fig. 3B).The values shown in Fig. 3B were used as the non-specific binding background of each cell line and were denoted as 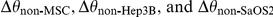 respectively for later analysis. On the contrary, in the group with OB-cadherin antibody coating, the angle shift of SaOS2 cells were distinguished from the Hep3B and the MSCs at day zero. This result demonstrated that SaOS2 has significantly higher OB-cadherin expression on cell surfaces, but none on Hep3B and on MSCs without osteogenic induction.

**Figure 4:**
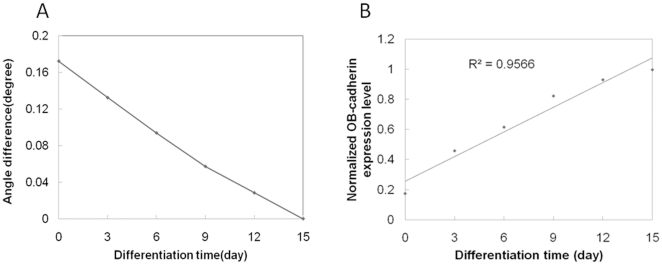
Angle shift difference during osteogenic differentiation of MSCs. (A) The angle difference, 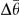, as a function of culture time. (B) OB-cadherin expression normalized to SaOS2 during differentiation.

Since MSCs that express more OB-cadherin on the cell surface had a better opportunity to be captured by the coated antibodies; this results in an increment of refractive index in the detection region, indicated by the angle shift. Therefore, the resonance angle 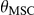 with a larger value would be obtained. The measurement was first conducted by the injection of the MSCs into the cell chamber. After the binding of MSCs reached the saturation status, PBS was used to wash out the MSCs followed by the injection of SaOS2 cells to measure the number of the active binding sites expressed in the SPR angle shift. A typical curve of SPR angle shift over the entire experimental procedure was demonstrated in Fig. 1C. In order to investigate the relative expression of OB-cadherin compared to the OB-cadherin positive SaOS2 cells, we used the angle shift obtained from SPR measurements to calculate the angle difference between the cells. As expression levels become similar, the angle difference will approach zero. Using the resonance angle before the injection of the MSCs as a reference value, denoted by 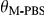, to subtract the value of the non-specific binding, 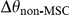, the amount of OB-cadherin expression on the membranes of the MSCs is then proportional to the shift of the resonance angles as 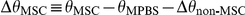. During each test, the different Au chip may have different number of active binding sites formed by anti-OB-cadherin, this number of active binding sites on the Au chip was measured by the angle shift starting from the injection of SaOS2 and ending at the saturation of the cell binding. The calculation can be formulated as 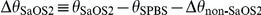. In the experiment, the relative value of the SPR angle shifts between MSCs and SaOS2 cells, indicated as OB-cadherin expression level 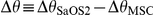, was normalized for data analysis. This normalization eliminated the variation of anti-OB-cadherin coating for each measurement. When the duration of osteogenic induction increased, the experimental value of angle difference (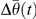) from MSCs subjected to different culture durations gradually approached to zero after 15-days (Fig. 4A).The interval of the MSC culture time for this measurement is three days until OB-cadherin levels on MSCs approached to the highest expression level and bound to anti-OB-cadherin antibodies with the same binding strength as SaOS2. This result suggested that both cell types are similar in surface OB-cadherin expression on the 15th day. Furthermore, when the angle difference 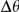 was normalized to 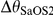 and denoted as 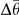, this normalization eliminated the variation of anti-OB-cadherin coating for each measurement. This quantity 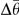 represents the complement of OB-cadherin expression. Therefore, the amount of OB-cadherin can be expressed as 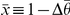. The experimental value of OB-cadherin expression level 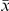 from MSCs subject to different culture durations was calculated. As expected, this angle difference approached to one after 15 days when the duration of osteogenic induction increased (Fig. 4B). The analysis showed that the expression level of OB-cadherin increased linearly with the increase of the culture time (days). By using this expression level curve as a standard, we could easily predict the maturation level of unknown differentiated live cells.

## Discussion

In this study, we developed a real-time, cell-preserving, and quantitative measurement method based on the surface plasmon resonance bio-sensing technique to characterize the maturation of osteoblasts by selective binding of a cell surface molecule, OB-cadherin, which is specifically and highly expressed in differentiating osteoblasts. With pre-coated antibodies on an Au chip, alteration of SPR angle shift could be accurately measured and analyzed. From the data analysis, it was found that the OB-cadherin expression level was increased as the culture time increased. The expression level obtained in the SPR biosensing method correlated with the result obtained by the conventional method of Western blot, but with much higher sensitivity at nanogram levels [29]. This new development offers a live cell tool to accurately define the different stages of osteogenic differentiation in live cells. Moreover, although the measurement was performed on osteoblast maturation, similar approaches can be applied in future studies to quantify the expression of OB-cadherin in tumor cells to predict their invasiveness and to also use other surface markers to monitor differentiation in other stem cell types [30].

In comparison with the results shown in Fig. 2C and Fig. 4B, the OB-cadherin expression level obtained by the Western blot assay in the early culture period did not match that obtained by the SPR biosensor, especially before the 9^th^ day of osteogenic differentiation. As shown in Fig. 2B, OB-cadherin expression was undetectable before day 6, with relatively weak signals until day 12. It is evident that Western blot detection lacks the ability to account for the vast range difference in OB-cadherin expression. For example, OB-cadherin expression at day 6 can be enhanced by prolonged exposure to chemiluminescent reagents. However, this would also saturate the reaction of HRP and chemiluminescent reagents of the day 15 group, resulting in inaccurate quantification. This explains why the relative quantitative level of OB-cadherin obtained by Western blot did not coincide with the result obtained by the SPR technique, but only coincided qualitatively in trend of expression. In addition, the most valuable advantage of this technique is its detection sensitivity whereby differences in OB-cadherin expression could be quantitatively distinguished by SPR technique during early differentiation stages between day 0 and day 6 (Fig. 4A), and not by Western blot (Fig. 2C). This also suggests that SPR is more accurate than the conventional Western blot method. Most importantly, live cells can be analyzed, which is not possible for the conventional Western blot method.

In summary, this SPR protocol makes possible the quantitative evaluation of different cell surface molecules on live cells in a time-saving and cell-saving manner for future cell biology research.
